# Severe Bacterial Superinfection of Influenza Pneumonia in Immunocompetent Young Patients: Case Reports

**DOI:** 10.3390/jcm13195665

**Published:** 2024-09-24

**Authors:** Szymon Białka, Michał Zieliński, Magdalena Latos, Marlena Skurzyńska, Michał Żak, Piotr Palaczyński, Szymon Skoczyński

**Affiliations:** 1 Department of Anaesthesiology and Intensive Care, Faculty of Medical Sciences in Zabrze, Medical University of Silesia in Katowice, 41-803 Zabrze, Poland; szymon.bialka@gmail.com (S.B.); piotr.palaczynski@gmail.com (P.P.); 2 Department of Lung Diseases and Tuberculosis, Faculty of Medical Sciences in Zabrze, Medical University of Silesia in Katowice, 41-803 Zabrze, Poland; michal.zielinski1@interia.pl (M.Z.); sz.skoczynski@sum.edu.pl (S.S.); 3 Clinical Department of Anaesthesiology and Intensive Care, Independent Public Clinical Hospital No. 1., 41-800 Zabrze, Poland; marlena.skurzynska1998@gmail.com; 4 Student Scientific Society at the Department of Anaesthesiology and Intensive Care, Faculty of Medical Sciences in Zabrze, Medical University of Silesia in Katowice, 41-800 Zabrze, Poland; michal181297@gmail.com

**Keywords:** respiratory infections, lobectomy, influenza, intensive care, bacterial coinfection, unvaccinated, young patients, infectious diseases, viral infection

## Abstract

Influenza can lead to or coexist with severe bacterial pneumonia, with the potential to permanently damage lung tissue, refractory to conservative treatment in the post-COVID-19 period. It can lead to serious complications; therefore, annual vaccinations are recommended. This case series with a literature review pertains to two young female patients with an insignificant past medical history, who required emergency lobectomy due to bacterial complications after influenza infection. Urgent lobectomy proves to be a feasible therapeutic option for selected patients with pleural complications.

## 1. Introduction

Respiratory viruses have played a significant role in causing community-acquired pneumonia. It was reported that before the COVID-19 pandemic, such agents were detected in 10–30% of patients with the aforementioned condition [1]. Viral epidemiology in this aspect has changed with the occurrence of severe acute respiratory syndrome coronavirus 2 (SARS-CoV-2) [2]. A global pandemic was declared by World Health Organization (WHO) on 11 March 2020, and it remained an international public health emergency until May 2023 [3]. During the pandemic, various interventions were undertaken in order to mitigate the effects of the disease. Pre-emptive measures such as avoiding physical contact with infected individuals, wearing face masks, and aerosol elimination by proper air treatment proved effective, not only for limiting the spread of coronavirus but also by affecting the prevalence of remaining microbial agents [4]. For some viruses, the prevalence increased in the second half of pandemic when restrictions eased [2]. An increase in influenza infections among children along with a concomitant decrease in COVID-19 cases was observed in March 2022 and reported by a Romanian pediatric hospital [5]. This may serve as a warning that while restrictions are relaxed, we should keep being vigilant.

Rhinovirus/enterovirus were the predominant respiratory viruses (excluding SARS-CoV-2) during COVID-19 pandemic, with a pooled prevalence of 5.05%. Influenza virus followed in these statistics as the second most prevalent with 3.27%, with a significantly higher prevalence of influenza A than influenza B species observed [2].

Influenza virus has always been described as one of the most common causes of severe viral pneumonia. Moreover, due to its high mortality and rapid transmission, it has been associated with a significant healthcare burden [6,7]. It was estimated that influenza virus accounted for approximately 500,000 deaths each year before the COVID-19 pandemic, with the majority of the deceased being either less than 5 years old or more than 75 years old [8,9]. Significant antigenic drift due to a segmented genome allows the virus to cause seasonal outbreaks every year [9,10]. Furthermore, strain dominance differs depending on the region of the world, and it is closely monitored and reported by the World Health Organization [6].

## 2. Case Series

**Figure 1:**
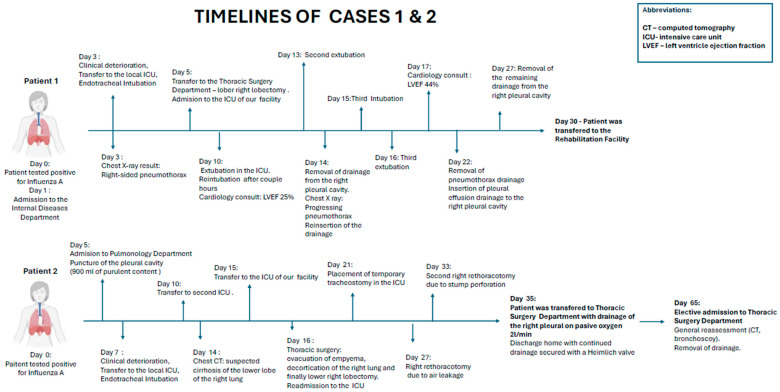
Timelines of the clinical courses of both patients.

This case series pertains to two young, female patients with an insignificant past medical history, who required emergency lobectomy due to bacterial complications after influenza infection. Detailed timelines of their clinical courses are presented in Figure 1. Both patients were treated at the same Polish facility in the spring of 2024. During the patients’ stay, a total of 25 patients were hospitalized in the ICU, including 17 with respiratory failure. Among them, 7 showed an exacerbation of chronic respiratory failure and 10 had acute respiratory failure. In our center, for the first time, we encountered cases presenting a need for surgical treatment due to complications of pneumonia in people so young. The doses of catecholamines were continuously adjusted depending on the current indications of hemodynamic monitoring. The Cardiac Index (CI), Stroke Volume Index (SVI), Preload—Global End-Diastolic Volume Index (GEDI), and Afterload—Systemic Vascular Resistance Index (SVRI) were continuously monitored using a hemodynamic monitoring device (HemoSphere, Edwards Lifesciences, Irvine, CA, USA). The Cardiac Function Index (CFI), Stroke Volume Variation (SVV), Pulse Pressure Variation (PPV), Extravascular lung Water Index (ELWI), and Pulmonary Vascular Permeability Index (PVPI) were also employed. Continuous veno-venous renal replacement therapy was performed with the Baxter PrisMax system using citrate regional anticoagulation. The parameters of acid–base balance, electrolytes (K^+^, Na^+^ Ca^2+^, total Ca, P^2+^), and kidney function were regularly monitored, and water balance was calculated twice a day. Based on the data obtained, the therapy settings were modified by regulating the flow: blood flow rate, flow before the blood pump, dialysate flow, replacement fluid flow, and CRRT dose.

Mechanical ventilation (ventilation with two-phase positive airway pressure) was used in accordance with the principles of lung protective ventilation so that the tidal volume was 4–6 mL/kg of the ideal body weight and the driving pressure (ΔP) < 14 cm H20.

### 2.1. Case 1

**Figure 2:**
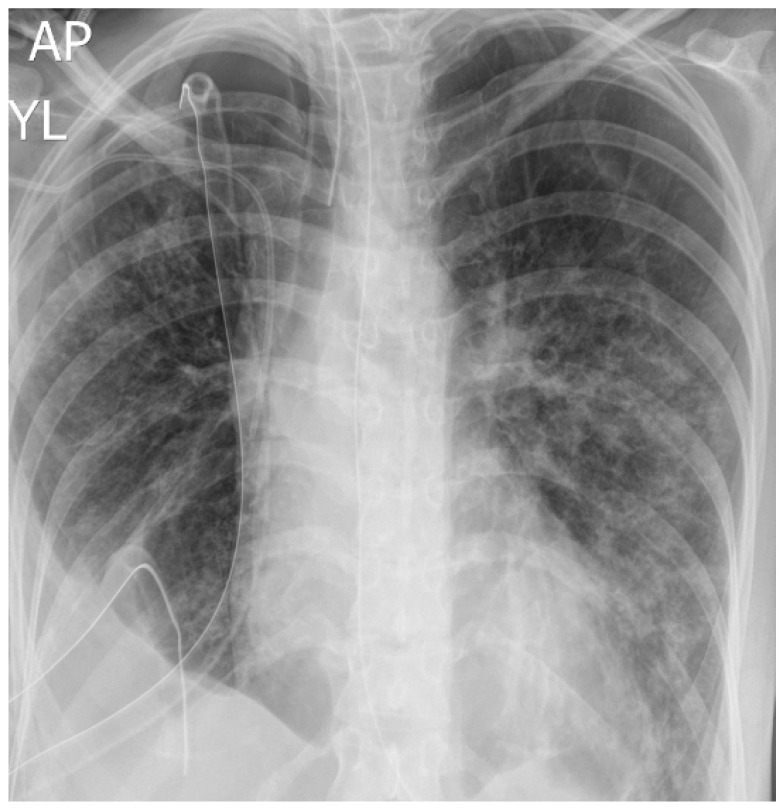
Chest X-ray of patient 1 at admission to the intensive care unit.

The first patient was a 36-year-old female who was initially hospitalized in the Department of Internal Diseases in the district hospital in a nearby city from day 1 to day 3 due to influenza infection. Her past medical history consisted of asthma and irritable bowel syndrome. She was admitted due to the worsening of respiratory symptoms despite antiviral treatment (oseltamiwir). On day 3, hemoptysis occurred, and decreased oxygen saturation was detected. Her clinical deterioration required admission to the ICU, where she was intubated and mechanically ventilated. Chest X-ray revealed right-sided pneumothorax, which was treated twice with pleural drainage—without the effect of lung expansion. She was then transferred for surgical treatment to the Thoracic Surgery Department in Zabrze due to suspected pleural empyema, lung abscesses, and pneumothorax with air leak. The chest X-ray performed at admission is presented in Figure 2.

After admission, bronchofiberoscopy was performed—sticky, mucopurulent secretion was aspirated and the BAL sample was collected for culture. After connecting to an active drainage system, the leakage of the breathing mixture was 4500 mL/min. It was changed to passive underwater drainage. Due to severe coagulation disorders, Octaplex was administered. The patient underwent emergency surgery due to ventilation problems and a severe septic condition. During the procedure, a right lower lobectomy was performed. After the procedure, she was transferred to the ICU in the same hospital in Zabrze for further treatment.

At post-operative admission to the ICU, the patient was in acute cardiorespiratory failure. Hemodynamic and vital signs’ monitoring was implemented. Mechanical ventilation, intensive anti-shock treatment, and targeted antibiotic therapy were continued, which were modified according to the results of microbiological tests. An assessment of the fungal pathogens was performed. A negative result was obtained for both patients. The detailed microbiological findings and antibiotic therapy are presented in Table 1. Based on the recruitment maneuvers performed, and determination of the optimal PEEP values and acid–base balance parameters, the mechanical ventilation settings were modified. Due to the increasing features of septic shock that did not respond to fluid therapy and norepinephrine infusion, argipressin infusion was added to the therapy. The treatment included antifungal drugs, analgesics, sedatives, mucolytics, antacids, steroids, inhaled bronchodilators, neuro- and hepatoprotective drugs, antiarrhythmics, diuretics, antithrombotic prophylaxis, vitamins, probiotics, enteral nutrition under the control of indirect calorimetry, and balanced water–electrolyte and acid–base therapies. Blood morphological deficiencies were supplemented. Anti-decubitus prophylaxis was used. Due to atelectasis, bronchofiberoscopy of the respiratory tract was performed several times. Due to arrhythmias and an increase in myocardial necrosis parameters as well as high NTproBNP values, the patient was consulted by a cardiologist. Cardiac ultrasound was performed—the valves showed no organic changes and vegetation, the left ventricle ejection fraction was 25%, and there was a normoechoic, round structure (suspected thrombus/vegetation) in the middle part of the right ventricle. Diagnosis of inflammatory cardiomyopathy with generalized hypokinesis was made. An infusion of dobutamine was added, which resulted in an improvement in hemodynamic parameters. In the following days, clinical improvement was noted. The infusion of vasoconstricting amines was gradually reduced. During hospitalization, the patient underwent cardiological consultation three more times (on the fifth, seventh, and twelfth postoperative day), which revealed an improvement in left ventricular function (LVEF approximately 44%) and the absence of vegetation previously visible in the right ventricular lumen. Respiratory improvement allowed for a reduction in mechanical ventilation support. On the fifth day of postoperative hospitalization, the patient was extubated with subsequent passive oxygen therapy.

**Figure 3:**
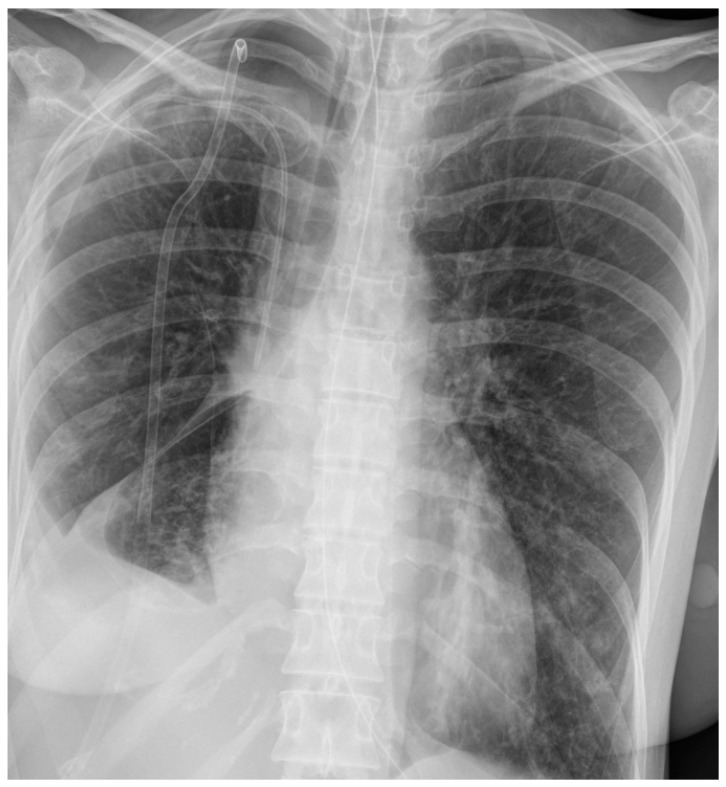
**Chest X ray of patient 1 during treatment**—progression of right-sided pneumothorax, which resulted in drainage of right pleural cavity.

After extubation, despite intensive kinesiotherapy, the patient presented an ineffective cough, leading to the accumulation of secretions in the respiratory tract and increasing respiratory effort with desaturation. The patient was intubated and mechanical ventilation was started. After intubation, a significant amount of serous content was aspirated from the airway. Bronchofiberoscopic cleaning of the bronchial tree performed on subsequent days revealed a patent bronchus intermedius and residual mucous content, which was aspirated. On the eighth day, the patient was extubated again, followed by passive oxygen therapy via a face mask. Due to repeated respiratory areflexia, secretion retention in the respiratory tract, and increased respiratory effort and desaturation, the patient was reintubated after approximately 38 h and mechanical ventilation was started. In the double drainage of the right pleural cavity, a decreasing value of leakage of the respiratory mixture was observed, and as a result the drain from the right pleural cavity was removed on the ninth day. Due to the progression of the right-sided pneumothorax visible in the follow-up chest X-ray, the patient was consulted by a thoracic surgeon again and drainage of the right pleural cavity was performed to observe the leakage of the respiratory mixture. The aforementioned chest X-ray is presented in Figure 3.

On the eleventh day of stay, after a thorough cleaning of the bronchial tree and meeting the necessary criteria, the patient was extubated again with subsequent passive oxygen therapy. In a follow-up ultrasound examination, the presence of free fluid in the right pleural cavity was observed. On the seventeenth day of ICU stay, and as a result of a thoracic surgical consultation, the drainage from the apex of the right lung was removed due to the lack of leakage of the respiratory mixture, and a Seldinger drain was placed to decompress the free fluid (in ultrasound >50 mm). On the twenty-third day, the drain from the right pleural cavity was removed. During the subsequent thoracic surgery consultation, a follow-up chest X-ray confirmed that the lungs had expanded and the patient did not require any surgical intervention. Histopathological findings from the resected lobe are presented in Table 1. Due to the symptoms of significant muscle weakness, mainly manifested by swallowing and breathing disorders, the patient was consulted by a neurologist—generalized muscle adynamia was diagnosed and blood was taken to determine the level of antibodies against Ach receptors (the result was negative). Pyridostigmine was added to the pharmacotherapy, with a marked improvement in the patient’s condition.

At this point, the patient was conscious, in full logical contact. She complied with quadriplegic commands with dominant muscle weakness in the lower limbs and could perform spontaneous breathing through natural channels with periodically applied passive oxygen therapy through a nasal cannula 1–2 L/min. The patient then returned to the Pulmonology Department of the District Hospital in a nearby city for treatment continuation.

### 2.2. Case 2

Another case pertains to a 35-year-old female patient who was transferred to our facility from the ICU of the Medical University Hospital in Katowice due to suspected cirrhosis of the right lower lobe. In the referral department, the patient was hospitalized due to acute cardiorespiratory failure due to pneumonia of mixed etiology (influenza A virus with secondary *S. pyogenes* infection) complicated by pleural empyema.

On day 0, the patient had a positive swab test for influenza virus. She began taking oseltamiwir instantly. However, her condition deteriorated to the point of that she admitted to the pulmonology department in a nearby city on day 5, where she underwent puncture of the pleural cavity (900 mL of purulent content was obtained). During her stay, she started developing symptoms of septic shock. On day 7, she was admitted to the ICU of the local hospital. At the beginning, she required high-flow nasal oxygen therapy, but her condition worsened. She was intubated with subsequent mechanical ventilation in place. She was then transferred to the Medical University Hospital in Katowice and admitted in critical condition to the ICU, with profound respiratory failure, hypoxemia, and hypercapnia despite invasive ventilation with FiO2 1.0. Intensive, multidirectional treatment was used (mechanical ventilation, circulatory support with catecholamine infusion, empirical and then targeted antibiotic therapy, RRT-CVVHDF with CytoSorb, right pleural drainage), achieving an initial stabilization of the patient’s general condition, improvement in ventilation conditions, reduction in the number and dose of catecholamines, and a temporary decrease in inflammatory parameters. On the 14th day, there was another increase in inflammatory parameters, and a follow-up CT scan of the chest revealed an increase in fluid in both pleural cavities. The consulting thoracic surgeon suspected cirrhosis of the lower lobe of the right lung, and a preliminary positive qualification for right lower lobectomy was made.

**Figure 4:**
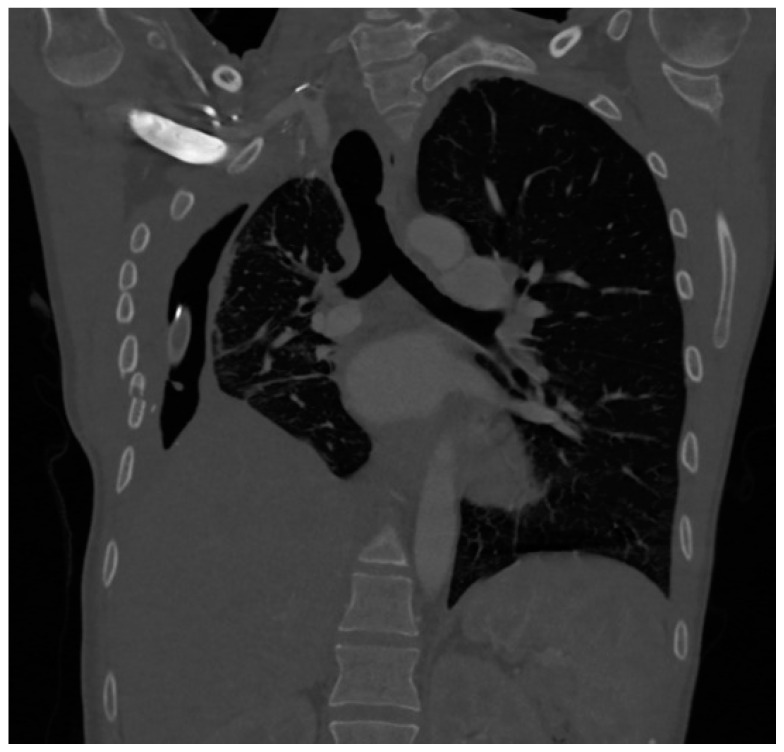
CT scan of the patient 2 at admission to the ICU.

The patient was then transferred to the ICU in our facility. Cultures obtained from her nasal swab as well as her anal swab were positive for Klebsiella pneumoniae. The remaining microbiological findings as well as her antibiotic therapy are presented in Table 1. She had also been surgically evaluated and underwent evacuation of empyema, decortication of the right lung, and finally lower right lobectomy, on the 16th day of hospitalization. She was transferred back to the ICU with drainage of the right pleural cavity. A CT scan image is presented in Figure 4.

Her pleural fluid and blood cultures were positive for *Klebsiella pneumoniae*. Her condition improved slightly, but extubating was still not feasible. On day 21, temporary tracheostomy was performed. Further microbiological assessments were positive for *K. pneumoniae*, but also for *Acinetobacter calcoaceticus*–baumannii and *Streptococcus pyogenes*. The patient needed a rethoracotomy twice. The first one was performed on day 27 as air leakage was found from the upper lobe parenchyma at the back, near the upper lobe bronchus. The second one pertained to suturing of the bronchial stump due to perforation and took place on the 33rd day. Purulent tissues of the right pulmonary hilum were observed and managed. During the ICU stay, she underwent cardiac assessment. Cardiac ultrasound did not reveal any significant abnormalities. She was then transferred to the thoracic surgery department for further treatment. On transfer day, the patient was conscious, mobilized, and upright, and respiratory rehabilitation was continued. With passive oxygen therapy of 2 L/min, there was a reduced vesicular sound over the lung fields at the base of the left lung and over the entire right lung field. Drainage of the right pleural cavity with air leak was maintained. After observation, she was discharged home with continued drainage secured with a Heimlich valve. She was again electively admitted for follow-up diagnostics, including CT scan, bronchoscopy, and general reassessment after a month. Significant improvements in lung aeration and low inflammation parameters were observed. The drain was removed. She was discharged home in quite good general condition. Histopathological findings from the resected lobe are presented in Table 1.

## 3. Discussion

Recent WHO report pertaining to influenza virus in the European region shows a continuous predominance of influenza A. However, its proportion increased to 96% in the studied period of 2023–2024, compared with 72% in 2022–2023. Currently, influenza B is sporadically detected in Europe (4%) [6]. The relative frequencies of influenza A subtypes have also shifted, with A/H1N1 viruses increasing (74% A/H1N1 vs. 26% A/H3N2) compared to the previous season (61% A/H1N1 vs. 39% A/H3N2) [6]. The majority of countries have reported detections of co-circulation of A/H1N1 and A/H3N2, with predominance of the latter, as well as sporadic detections of influenza B/Victoria. What is more interesting, it was reported that B/Yamagata-lineage viruses may have become extinct after the COVID-19 pandemic, as their circulation has not been verified since March of 2020 [11]. This further emphasizes the changes in the microbiome in the post-pandemic world.

Influenza virus can lead to severe pneumonia, but its mortality is usually attributed to its complications, especially among patients with pre-existing pulmonary or cardiovascular conditions [8,9,12,13]. Patients with asthma, chronic obstructive pulmonary disease (COPD), and cystic fibrosis (CF) are more prone not to only to bacterial or fungal infection but also to developing acute respiratory distress syndrome (ARDS) [7]. Moreover, bacterial infection is a common cause of hospitalization among otherwise healthy individuals with influenza [9,12,14]. Bacterial co-infection during the 2009 H1N1 pandemic was associated with high mortality rates despite proper antibiotic therapy [12,15,16].

It has been documented that severe influenza syndrome may lead to a release of cytokines, which results in the dysregulation of the immune system regardless of secondary infection [15,17]. The mechanisms underlying post-viral bacterial infections include interactions between viruses, bacteria, and a patient’s immune system. Antiviral immune response triggered by the influenza virus can be associated with changes in the microbiome of the respiratory tract. It may change immune function to the point of actually enhancing the proliferation of pathogenic bacteria [15].

Among healthy people, microbial communities in the upper respiratory tract are more diverse in comparison to the its lower part. The microbiome of the lungs consists mostly of Bacteroidetes and Firmicutes (mainly *Prevotella*, *Veillonella*, and *Streptococcus*) [15,18]. Proper maintenance of the microbiome of the entire respiratory tract is crucial as bacterial colonization of the upper part is often considered to be the first step in the development of invasive bacterial infections, as well as bacterial infections, which follows respiratory viral infection [15,19]. It is reported that the upper respiratory tract microbiome may be enriched with Proteobacteria (i.a *A. baumanii*, *Pseuomonas* spp.); Firmicutes (i.a. *S. aureus*, *S. pneumoniae*); and *H. influenzae*, *M. catarrhalis,* and *K. pneumoniae* after confirmed influenza infection [20,21,22].

Despite the aforementioned microbiome changes, influenza virus can moderate a host’s immune response. It was reported that A/H1N1 has the ability to cause a cytokine storm that may be associated with ARDS and severe multi-organ failure [23]. Additionally, various types of white blood cells have also been shown to have a reduced phagocytic capacity during influenza infection [9].

What is more, immune system dysfunction can progress further during bacterial coinfection/secondary bacterial infections, as a synergic disruptive effect was reported, particularly with *S. aureus* and *S. pneumoniae* [24]. Mice models that were assessed following pneumonia in the lungs and mediastinal lymph nodes showed increased virus titers and bacterial cell counts, a decreased level of virus-specific immunoglobulins, and certain types of white blood cells. In the mediastinal lymph nodes of mice, a significantly decreased amount of germinal center B cells, T follicular helper cells, and plasma cells were detected in lethal cases of coinfection [24]. An unfortunate fatal coinfection of influenza and *S. aureus* was also reported in previously healthy 17-year-old, which confirmed the significant immunosuppressive abilities of both the A/H1N1 strain and bacterial agents [14].

The described characteristics may sometimes lead to a severe clinical course of the disease among patients with comorbidities, but they can also happen among young and relatively healthy people. Patients with respiratory infection due to influenza virus with bacterial coinfection/secondary infection may progress to a clinical state that merits admission to an intensive care unit (ICU). The majority of admitted patients demonstrate pulmonary involvement requiring mechanical ventilation. Moreover, SAPS II score at admission, need for vasoconstricting drugs, and endotracheal intubation within the first 48 h were assessed as significantly increasing the chance of death. Despite proper treatment, ICU mortality is mostly impacted by age (<65 years), history of cancer disease, severity of ARDS, and the presence of bacterial coinfection [25].

Influenza and bacterial coinfection or bacterial pneumonia secondary to influenza can result in significant pulmonary complications, which have mostly been assessed and studied during A/H1N1 outbreaks. Back then, 46.6% of patients had a documented bacterial co-infection, which among all critically-ill patients was 20–32% [7]. Data from 23 French ICUs indicate that they were mostly caused by Streptococcus pneumoniae (54%) or Staphylococcus aureus (31%) [26]. Instances of *K. pneumoniae*, *S. oralis*, *H. influenzae*, *M. catarrhalis,* and *L. pneumophila* were also reported in a Korean study [27]. Coinfection with *S. aureus* or pneumococcus pneumoniae strains during ICU stay was not correlated with mortality. Therefore, it is advised to diagnose the presence of bacterial coinfections at admission to a hospital as well as in the ICU to properly and timely implement effective antibiotic therapy [25].

The majority of data pertaining to influenza viruses focuses on the more prevalent type A. However, it is important to acknowledge a series of case reports of Streptococci and Staphylococcal pneumonia with concomitant influenza B infection, which have since demonstrated potential morbidity and mortality in adults [7]. In the northern hemisphere during the 2017–2018 season, influenza B virus predominantly caused infections [28].

Pulmonary complications over the course of infectious disease may result in focal bronchiectasis or cavitary infectious lung disease, which may require surgical resection. The majority of patients benefiting from such treatment (mostly lobectomy) with the aforementioned lesions were operated on due to active or recurrent mycobacteriosis [29]. Furthermore, pneumonia may progress to necrotizing lung infection or lung abscess, or it may present as permanent atelectasis with pneumothorax [30]. The most common pathogens cultured from lung tissue in a study pertaining to acute necrotizing lung infections requiring thoracic intervention were *Streptococcus pneumoniae* (15/35 cases) and *Staphylococcus aureus* (11/35 cases), with some cases of *Pseudomonas aeruginosa*, *Klebsiella,* and *Haemophilus* species [30]. In other words, these are the bacterial agents mostly associated with post-viral bacterial infections/viral and bacterial coinfections in course of influenza. It seems that patients with an unfavorable course of influenza may develop pulmonary complications so severe that they require thoracic surgery.

During the COVID-19 pandemic, post-viral bacterial infections/viral and bacterial coinfections over the course of SARS-CoV-2 delivered multiple parallel examples of serious pulmonary complications that require surgical management [31].

Influenza virus poses a global threat each year by causing infections associated with high morbidity and mortality. Public health preventative measures, such as seasonal influenza vaccines, have been routinely used for many decades [32,33,34]. Vaccine strain selection must be conducted months before vaccine distribution; therefore, it can be challenging [33,34]. A study by Gross et al. confirmed that influenza vaccination is effective in the reduction of pneumonia, hospitalization rate, and death if the vaccine strain and epidemic strain are similar, especially among the older patients [35]. It was associated with less outpatient visits for pneumonia. It also decreased the frequency of hospitalization [7]. Vaccination against other prevalent pathogens may affect the type of bacterial coinfection, as the rate of S. pneumonia seems to be decreasing, likely due to vaccination, and that of H. influenza and P. aeruginosa are on the rise according to observations [7,36]. Both patients have never received their influenza vaccinations by choice. In adults (>18 years old), influenza vaccine effectiveness was 38% (95% CI 30–45%) in those with high-risk conditions versus 44% (95% CI 38–50%) among generally healthy individuals [32].

Patient number 1 had a prior respiratory condition (asthma), which can be exacerbated by influenza. Her clinical course was so severe that she was admitted to the ICU. Verdier et al. performed an analysis of mortality factors among patients hospitalized in the ICU due to influenza. They fortunately demonstrated that asthma does not increase the risk of death among their studied group [25]. It has not been established whether influenza vaccination has the ability to prevent asthma exacerbations [37]. However, using a systematic review and meta-analysis, Vasileiou et al. assessed that vaccination was associated with a 59–78% reduction in asthma episodes, leading to emergency visits or hospitalizations [38]. Patient number 1 also suffered from cardiovascular complications of influenza, with her LVEF decreasing to 25%. It is worth noting the significant association between influenza vaccine and a reduced risk of cardiovascular events, including myocardial infarction [7].

Both female patients were in their 30s and were relatively healthy. They both acquired influenza in the spring of 2024. This means that they became sick after the COVID-19 pandemic. The available literature provides only one similar case of a young, relatively healthy woman with severe pneumonia of H1N1 origin with bacterial superinfection with Staphylococcus aureus. Her clinical course was characterized by a high level of interleukine-6 and a rapid and lethal course [14].

Some studies suggest that susceptibility to influenza virus infection can depend on the host. Susceptible people may have impaired intracellular controls of viral replication, defective interferon responses, or defects in cell-mediated immunity, with an increased systemic inflammation baseline [39]. Other studies indicate that individuals under stress have weakened immunity, making them more prone to more severe courses of viral infection, as reported by a study of stress-induced susceptibility to influenza with the use of corticone among mice [40]. It was also noted that childhood seems to be a crucial time to develop immunity to influenza, as our first exposure to the influenza antigen determines the quality of lifelong antiviral immunity [41]. This is supported by the fact that older patients experienced lower rates of A/H1N1 infection during the 2009 pandemic, as they underwent exposure to A/H1N1 antigens in 1918–1919 during the Spanish flu pandemic [42].

It is difficult to assess which of the aforementioned factors pertains to our patients, as both their cases are described retrospectively. Although individual assessments of influenza susceptibility sound promising, they are currently not a standard of care, especially pre-emptively. It is worth noting that both patients experienced severe bacterial complications, particularly in their lung tissue. The decision to perform lobectomy among patients that young proved to be appropriate in the clinical context. However, the available literature is lacking in this aspect. The majority of publications pertaining to lung resection (pneumectomy or lobectomy) as a treatment measure for infection pertains tuberculosis and mycobacterium other than tuberculosis [29,43,44].

Mitchel et al. reported that the most common non-mycobacterial agent associated with surgical treatment among 171 patients with bronchiectasis or cavity lung disease was *P. aeruginosa* [29]. Contrary to the emergency cases presented here, all patients underwent elective targeted anatomic resection to remove damaged lung parenchyma. The paper proves that lobectomy can be a feasible treatment for the selected group of patients. They reported 0% operative mortality, with 5.6% of patients experiencing prolonged air leak (the most common noted complication) [29]. Patient number 2 also had complicated prolonged air leak, managed with drainage, which was ultimately treated with good outcome. Pneumectomy was also reported as a therapeutic option for infectious lung disease by Blyth, with tuberculosis being the main indication (72% of cases) [44]. This author also describes an emergency pneumectomy performed on a 57-year-old male with tuberculosis due to hemoptysis with unfavorable outcome.

## 4. Conclusions

Influenza can lead to or coexist with severe bacterial pneumonia, with the potential to permanently damage the lung tissue, refractory to conservative treatment. Urgent lobectomy proves to be a feasible therapeutic option for selected patients with the aforementioned complications.

## Figures and Tables

**Table 1:** Microbiological assessment, antibiotic treatment, and histopathological findings of both patients.

Culture Origin	Patient 1	Patient 2
Anal swab	*Enterococcus faecalis HLAR, VRE*	*Escherichia coli OXA-48*
	*Staphylococcus haemolyticus*	*Eschierichia coli ESBL*
	*Pseudomonas aeruginosa*	*Klebsiella pneumoniae KPC*
		*Pseudomonas aeruginosa*
		*Candida* spp.
Nasal swab	*no data*	*Klebsiella pneumoniae*
Pleural fluid culture	*Candida dubliniensis*	*Candida glabrata*
		*Klebsiella pneumoniae KPC*
Urine culture	*Pseudomonas aeruginosa*	*no data*
Blood culture	*Staphylococcus epidermidis*	*Klebsiella pneumoniae KPC*
PCR respiratory panel	*Staphylococcus* spp.	*Acinetobacter calcoaceticus-baumannii*
	*Staphylococcus epidermidis*	*Klebsiella pneumoniae*
		*Streptococcus pyogen*
Antibiotic treatment during intensive care stay	Colistin 3 × 3 million iv (for 10 days), colistin 3 × 2 million inhalations (for 10 days), meropenem 3 × 1 g iv (for 8 days), tigecycline 2 × 50 mg iv (for 11 days), ampicillin + sulbactam 1 g + 0.5 g × 4 (for 10 days), avibactam + ceftazidime 2 g + 0.5 g × 3 iv (for 10 days), amikacin 1.2 g × 1 iv (for 7 days).	Clindamycin 900 mg × 3 iv (for 9 days), vancomycin 2 times a day iv according to blood levels, meropenem 3 × 1 g iv (for 5 days), penicillin 24 mL iv in continuous infusion (for 8 days).
Histopathological findings from the resected lobes	Purulent bronchopneumonia with foci of parenchymal necrosis and formation of abscesses, fibroblastic foci in the lumen of the alveoli, and purulent pleuritis.	Purulent lobar pneumonia with foci of necrosis, purulent pleurisy, and purulent inflammation around the vessels.

Legends: ESBL—extended-spectrum beta-lactamases, HLAR—high-level aminoglycoside resistance, KPC—*Klebsiella pneumoniae* carbapenemase, OXA-48—oxacillinase 48, VRE—vancomycin-resistant enterococcus.

## Data Availability

Data is unavailable due to privacy of the patients.
